# AS101 Prevents Diabetic Nephropathy Progression and Mesangial Cell Dysfunction: Regulation of the AKT Downstream Pathway

**DOI:** 10.1371/journal.pone.0114287

**Published:** 2014-12-04

**Authors:** Itay Israel Shemesh, Benaya Rozen-Zvi, Yona Kalechman, Uzi Gafter, Benjamin Sredni

**Affiliations:** 1 Safdié Institute for AIDS and Immunology Research, The Mina and Everard Goodman Faculty of Life Sciences, Bar-Ilan University, Ramat Gan, Israel; 2 Department of Nephrology and Hypertension, Rabin Medical Center, Petah-Tikva, Israel; 3 Sackler School of Medicine, Tel Aviv University, Tel-Aviv, Israel; National Centre for Scientific Research “Demokritos”, Greece

## Abstract

Diabetic nephropathy (DN) is characterized by proliferation of mesangial cells, mesangial expansion, hypertrophy and extracellular matrix accumulation. Previous data have cross-linked PKB (AKT) to TGFβ induced matrix modulation. The non-toxic compound AS101 has been previously shown to favorably affect renal pathology in various animal models and inhibits AKT activity in leukemic cells. Here, we studied the pharmacological properties of AS101 against the progression of rat DN and high glucose-induced mesangial dysfunction. In-vivo administration of AS101 to Streptozotocin injected rats didn’t decreased blood glucose levels but ameliorated kidney hypotrophy, proteinuria and albuminuria and downregulated cortical kidney phosphorylation of AKT, GSK3β and SMAD3. AS101 treatment of primary rat glomerular mesangial cells treated with high glucose significantly reduced their elevated proliferative ability, as assessed by XTT assay and cell cycle analysis. This reduction was associated with decreased levels of p-AKT, increased levels of PTEN and decreased p-GSK3β and p-FoxO3a expression. Pharmacological inhibition of PI3K, mTORC1 and SMAD3 decreased HG-induced collagen accumulation, while inhibition of GSK3β did not affect its elevated levels. AS101 also prevented HG-induced cell growth correlated to mTOR and (rp)S6 de-phosphorylation. Thus, pharmacological inhibition of the AKT downstream pathway by AS101 has clinical potential in alleviating the progression of diabetic nephropathy.

## Introduction

Diabetes is the leading cause of end-stage renal disease, accounting for over 50% of patients new to dialysis in developed countries, and is the most common and serious complication of diabetes [1]. Available therapies, including adequate glycemic control and anti-hypertensive therapy, slow down but do not halt the progression of renal dysfunction in diabetic nephropathy (DN) [2]–[4]. It is therefore necessary to develop novel therapeutic agents that target the major pathological mechanisms of this condition. DN encompasses distinct pathologies including discrete structural alterations, including renal hypertrophy, thickening of basement membranes, and progressive glomerular accumulation of extracellular matrix (ECM) components, which ultimately results in irreversible renal fibrosis. Hyperglycemia is an indispensable prerequisite to the pathogenesis of diabetic renal disease [5], and its implications are initially evident in mesangial cell alterations. Previous studies showed that raising glucose concentrations in mesangial cell culture media from 100 to 450 mg/ml (30 mM) resulted in early cell proliferation, followed by an antiproliferative hypertrophic effect and extra ECM accumulation [6], [7].

Diabetic induced glomerulosclerosis is caused by accumulation of ECM proteins in the mesangial interstitial space, resulting in fibrosis manifested by either diffuse or nodular changes [6]. The most common matrix proteins detected are collagen type I, III, IV, and fibronectin [7], which accumulates due to increased synthesis by mesangial cells and reduced degradation by metalloproteinases [8]. Regarding the molecular mechanisms accelerating DN progression, including the onset of mesangial collagen accumulation, TGFβ has already been identified as a master regulator cytokine, mediating these effects [9], [10]. The intracellular SMAD pathway, which transduces TGFβ signaling, is accountable for collagen type 1 transcription and integrity [11]. However, intervention of other pathways, supporting TGFβ/SMAD3 signaling, might change the fibrotic outcome. For instance, the PI3K/AKT pathway has been described as a crucial pathway promoting TGFβ – induced collagen type 1 accumulation [12]. Moreover, there is evidence of dependence between HG induced collagen type 1 accumulation in mesangial cells, and PI3K/AKT activity [13], [14]. These findings suggest a necessary cross-talk between the various pathways resulting in mesangial fibrotic pathology. In addition, the role of AKT signaling in mediating mesangial deregulation does not conclude in collagen accumulation alone, and other properties such as viability and proliferative effects also contribute to AKT activity in various models [15], [16].

The nontoxic ammonium trichloro(dioxoethylene-o,o’)tellurate (AS101) first developed in our laboratory, has already been shown to have beneficial effects in diverse preclinical and clinical studies. Previous studies by our group demonstrated the ability of AS101 to decrease LPS-induced mesangial cell proliferation in-vitro. This was followed by another study demonstrating the inhibition of mesangial cell proliferation in an experimental mesangial proliferative glomerulonephritis in-vivo model, suggesting that AS101 can ameliorate the progression of inflammatory glomerulonephritis via inhibition of the IL10-STAT pathway [17], [18]. Thus, the effect of AS101 in prevention of non-diabetic renal failure was primarily attributed to its immune-modulating activity.

However, another possible mechanism through which AS101 exerts its molecular modifications was suggested by one of our most recent studies. AS101 downregulates AKT phosphorylation in cancerous leukemic cells via VLA-4 integrin inhibition, leading to reduction of PI3K/AKT signal transduction [19]. The role of PI3K/AKT in mesangial cell-mediated DN progression, and the ability of AS101 to inhibit AKT phosphorylation in cancer cells, led us to investigate the possibility of AS101-induced renal tissue protection under HG conditions, via modification of the PI3K/AKT pathway.

Here, we show that AS101 administration leads to the protection of kidney integrity in STZ injected rats. While blood glucose levels remained high, administration of AS101 prevented kidney hypertrophy, and reduced urine protein and albumin levels. In-vitro, HG-induced mesangial cell over proliferation, mesangial expansion, enlargement of cell size and collagen accumulation were all mitigated when cells were treated with AS101. Additionally, these cellular changes were all correlated with downregulation of AKT signal transduction pathways.

## Results

### AS101 prevents proteinuria and albuminuria without affecting blood glucose levels in diabetic rats *in-vivo*

**Figure 1:**
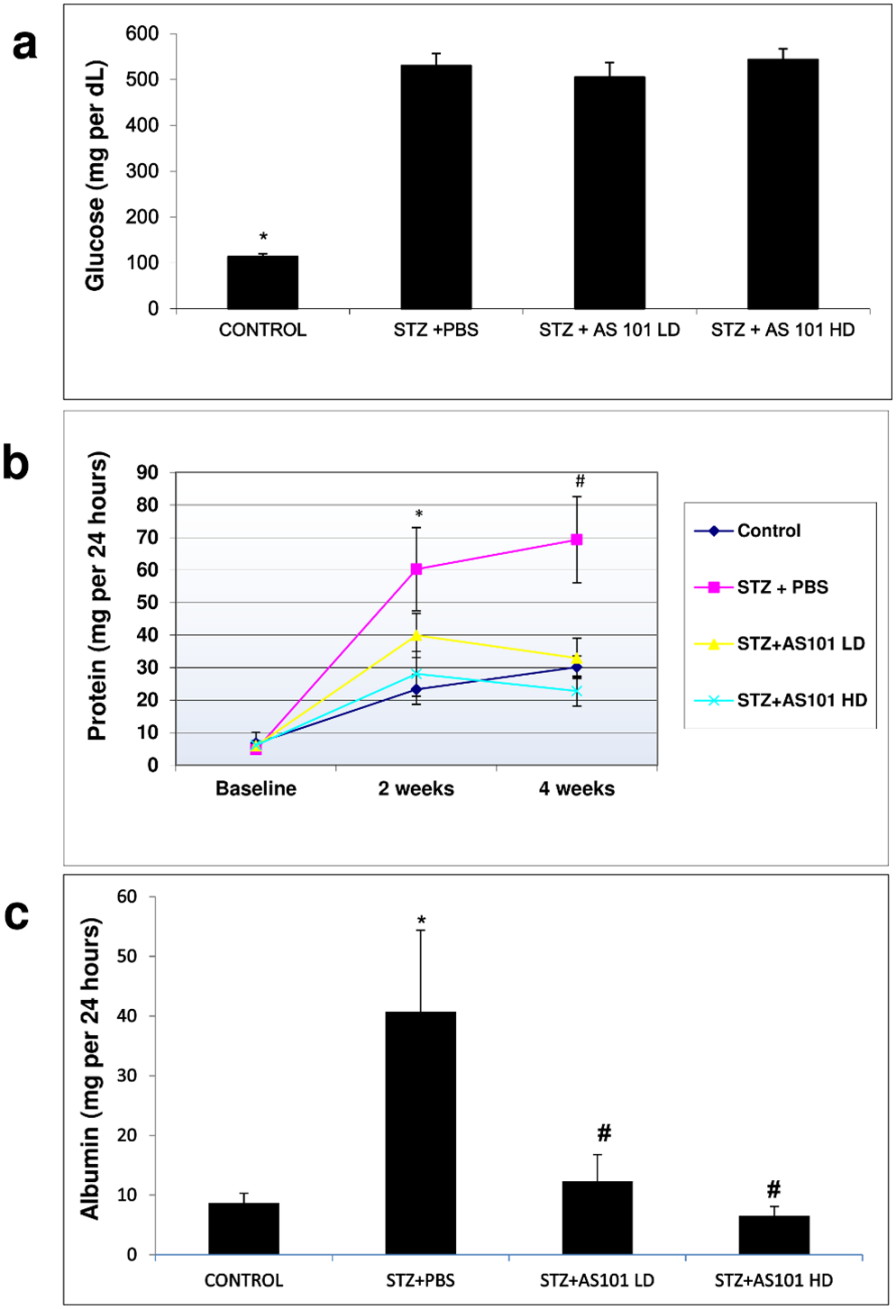
AS101 prevents proteinuria and albuminuria but does not affect blood glucose levels *in-vivo*. Diabetes was induced by single intraperitoneal injection of STZ (65 mg/kg). Mice were treated with AS101 at 0.5 mg/kg (Low dose - LD) or 1 mg/kg (High dose - HD) by intraperitoneal injection every other day. Control animals were treated with PBS alone. (a) Glucose levels were determined by Freestyle glucometer. *p<0.01 decrease vs. all other groups (n = 14 for Control; n = 12 for STZ+PBS; n = 15 for STZ+AS101 LD; n = 14 for STZ+AS101 HD). (b) Urine was collected by metabolic cage for 24 hours, and protein was determined by Bradford assay. *p<0.05 increase vs. STZ+HD and control groups. #p<0.01 increase vs. all other groups (n = 14 for Control; n = 12 for STZ+PBS; n = 15 for STZ+AS101 LD; n = 14 for STZ+AS101 HD). (c) Urine albumin was determined by ELISA. *p<0.05 increase vs. control. #p<0.05 decrease vs. PBS treated diabetic rats (n = 5 for Control; n = 6 for STZ+PBS; n = 9 for STZ+AS101 LD; n = 8 for STZ+AS101 HD).

One of the most well-defined markers for renal dysfunction is proteinuria. We therefore used proteinuria as a marker to characterize the effect of AS101 on DN progression in a rat model of Type 1 diabetes. Blood glucose at 4 weeks rose significantly in all of the Streptozotocin (STZ) treated rats without significant differences between the groups (Fig. 1a). Specifically, STZ+AS101 treated rats showed no difference in blood glucose levels compared to the STZ+PBS treated group. Suggesting that administration of AS101 has no effect on the diabetic state of the animals (Fig. 1a). Nevertheless, urine protein excretion at 2 weeks was significantly lower in STZ+AS101 given at high dose (HD; 1 mg/kg) compared to PBS treated diabetic rats (STZ+PBS). After 4 weeks, significant decrease was achieved in AS101 treated diabetic rats, at both low (LD; 0.5 mg/kg) and high doses (AS101 LD 33±6.1 mgr/24 h, AS101 HD 23±4.6 mgr/24 h) compared to PBS treated diabetic rats (69±13.2 mgr/24 h), With significant increase at both 2 and 4 weeks between PBS treated diabetic rats and control animals and no significant difference between AS101 treated and control animals (Fig. 1b). In addition, urinary albumin excretion at 4 weeks, were compatible to urinary protein results (control 12±6 mg/24 h, STZ+PBS 41±17 mg/24 h, STZ+AS101 LD 12±4 mg/24 h, STZ+AS101 HD 6±2 mg/24 h, Fig. 1c). These results indicate that AS101 administration can prevent the development of proteinuria under diabetic conditions in-vivo.

### AS101 prevents renal hypertrophy in STZ injected rats

**Figure 2:**
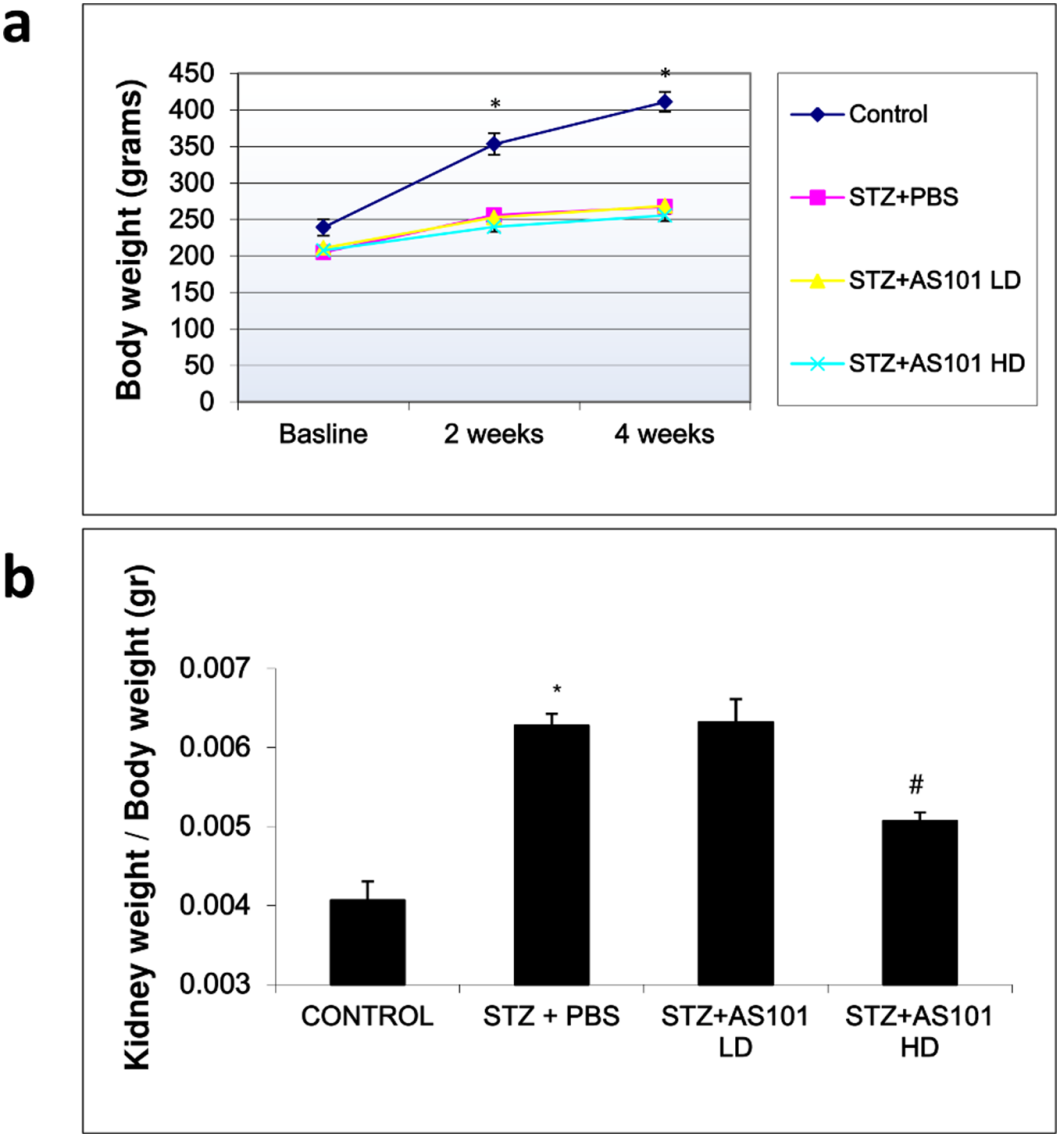
AS101 prevents renal hypertrophy in STZ injected rats. Diabetes was induced by single intraperitoneal injection of STZ (65 mg/kg). Mice were treated with AS101 at 0.5 mg/kg (LD) or 1 mg/kg (HD) by intraperitoneal injection every other day. Control animals were treated with PBS alone. (a) Total average body weight of each group was monitored, *p<0.01 decrease vs. all other groups (n = 14 for Control; n = 12 for STZ+PBS; n = 15 for STZ+AS101 LD; n = 14 for STZ+AS101 HD). (b) Kidneys were removed and weighed after 4 weeks and kidney weight was normalized to body weight. *p<0.01 increase vs. control group. #p<0.01 decrease vs. STZ+PBS group (n = 5 for Control; n = 7 for STZ+PBS; n = 9 for STZ+AS101 LD; n = 8 for STZ+AS101 HD).

Another characteristic feature of Diabetic nephropathy is kidney hypertrophy. Here, we calculated kidney weight (mg) in correction to body weight (gr) to assess kidney hypertrophy. As depicted in figure 2a, body weight changes in diabetic rats treated with PBS or AS101 were comparable, while Control rats had a significant increase in body weight vs. all other groups at both 2 and 4 weeks of treatment. Kidney hypertrophy as assessed by kidney weight (mg) normalized to body weight (gr) was significantly increased in the PBS treated diabetic rats (6.5±0.2) versus control rats (4±0.07). Treatment with AS101 at high dose abolished kidney hypertrophy, with significant decrease in kidney weight compared to PBS treated diabetic rats (STZ+AS101 HD 5.1±0.2, Fig. 2b). Although AS101 did not restore kidney weight to control levels, its administration at 1 mg/kg significantly decreased kidney weight compared to STZ injected rats, suggesting some beneficial effect against kidney hypertrophy.

### AS101 downregulates AKT, GSK3β and SMAD3 cortical kidney phosphorylation in-vivo

**Figure 3:**
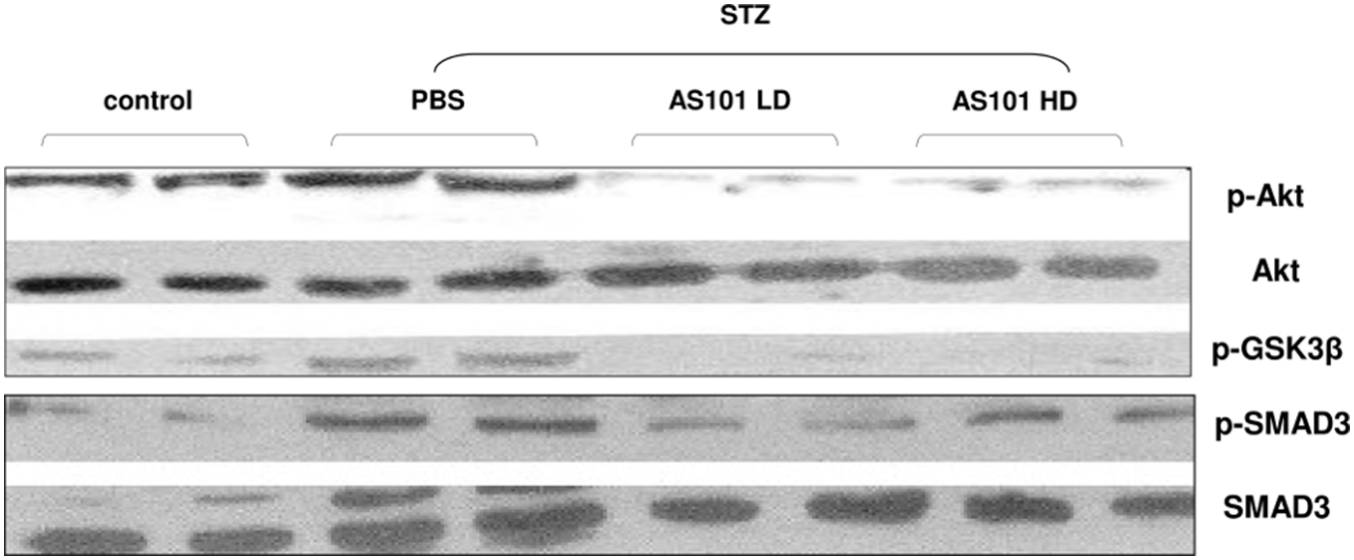
AS101 downregulates AKT, GSK3β and SMAD3 phosphorylation on cortical kidney cells in-vivo. Diabetes was induced by single intraperitoneal injection of STZ (65 mg/kg). Mice were treated with AS101 at 0.5 mg/kg (LD) or 1 mg/kg (HD) by intraperitoneal injection every other day. Control animals were treated with PBS alone. Western blot analysis of cortical tissue was performed 4 weeks after diabetes induction. Lysates were extracted for detection of p-AKT, p-GSK3β, and p-SMAD3 by western blot analysis. Total AKT and SMAD antibodies were used as a loading control. Results shown are from one experiment representative of three.

The beneficial effect of AS101 administration on progression of DN was next studied on the molecular level. To this end, molecular mechanisms mediating the diabetic effect were investigated on cortical tissue of treated vs. non treated rats. Western blot analysis of cortical tissue revealed increased p-AKT levels in PBS treated diabetic rats compared to control rats. Treatment with AS101 at both doses of 0.5 mg/kg (LD) and 1 mg/kg (HD) prevented the elevation of AKT phosphorylation levels (Fig. 3). AS101 treatment also prevented phosphorylation of GSK3β and SMAD3 compared to PBS treatment in diabetic rats (Fig. 3). This downregulation by AS101 of the phosphorylation of key factors including p-AKT, p-GSK3β and p-SMAD3 in cortical kidney tissue indicates its wide-spread effects. Moreover, these phosphorylation changes suggest an essential role of AKT signaling in orchestrating the spectrum of effects on the tissue (hypertrophy, proteinuria, and albuminuria).

Nonetheless, its pleiotropic effects on the tissue, led us to investigate whether AS101 has the same effect on AKT downstream signaling in specific glomerular cells such as mesangial cells which have a pivotal role in the progression of DN [20], [21].

### AS101 attenuates high glucose induced mesangial cell proliferation

**Figure 4:**
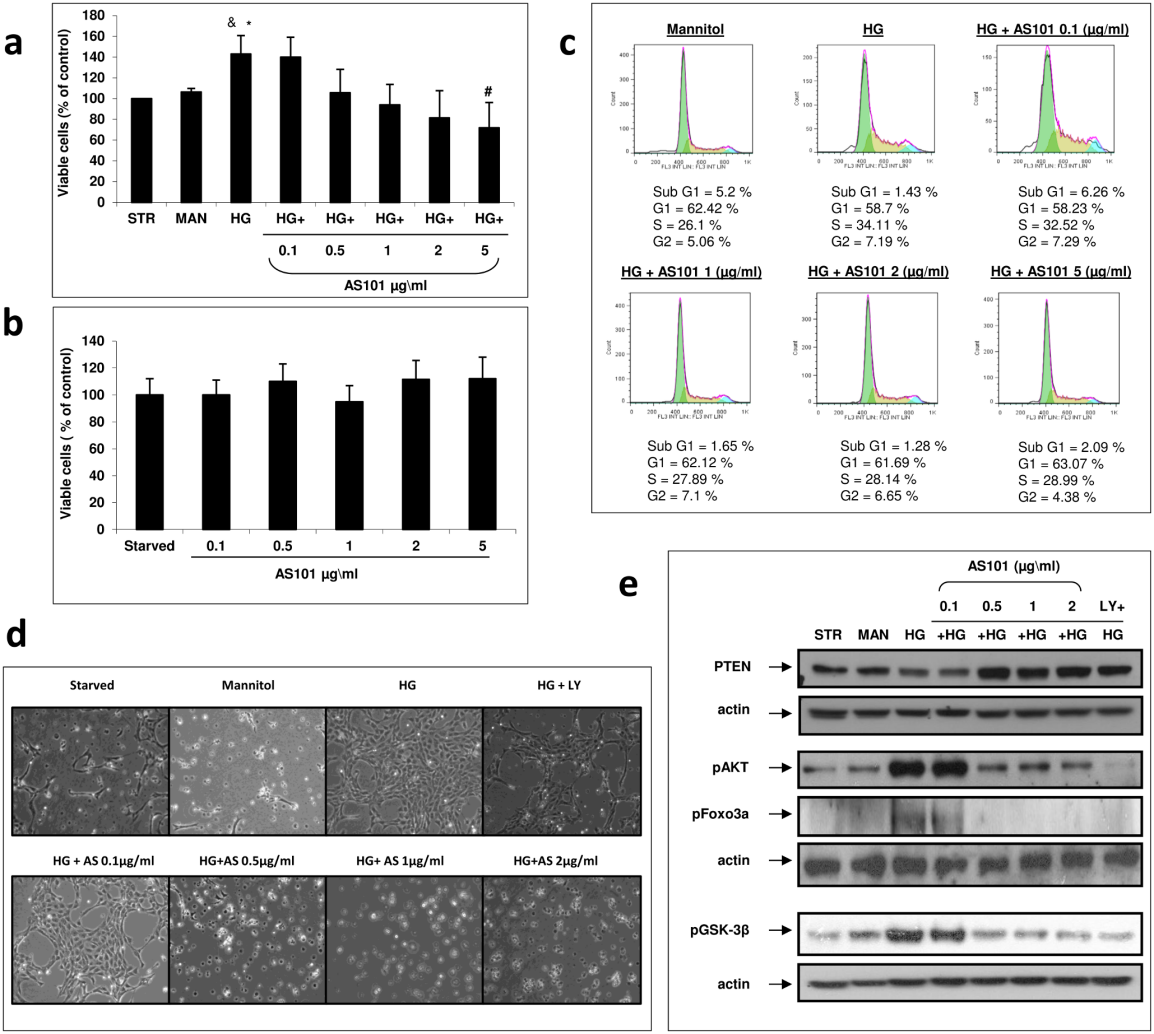
AS101 attenuates high glucose induced mesangial cell proliferation. Mesangial cells were cultured in DMEM without serum for 24 h. Cells were then transferred to fresh DMEM without serum in the presence or absence of HG treatment (30 mM). For osmotic control, cells were treated with Mannitol (24.5 mM). Medium for the serum deprived control (Starved) or the osmotic control (Mannitol) contained normal levels of glucose (5.5 mM). (a) Cells treated with HG were supplemented with different concentrations of AS101 (0.1, 0.5, 1, 2, 5 µg/ml). After 24 h, XTT assay was performed. OD levels were compared to control which was normalized to 100%. Results represent mean±SEM from three experiments. #p<0.05 decrease vs. HG. *p<0.05 increase vs. starved. &p = 0.057 increase vs. mannitol. (b) Cells treated with Normal glucose levels were supplemented with AS101 at the concentrations indicated in panel a. After 24 h, XTT assay was performed. OD levels were compared to control which was normalized to 100%. Results represent three experiments. (c) Cells treated with HG were treated with different concentrations of AS101 (0.1, 1, 2, 5 µg/ml). After 48 h of treatment, cells were stained with PI buffer; cell cycle analysis was performed by FACS to determine the level of cell accumulation in each cell cycle phase: sub-G1, G1, S, G2. Results represent two experiments. (d) Cells treated with HG were treated with different concentrations of AS101 (0.1, 0.5, 1, 2 µg/ml) or LY294002 (50 µM). After 24 h, mesangial cell expansion was examined by light microscopy (X40). Results shown are from a single experiment representative of seven. (e) Cells treated with HG were treated with different concentrations of AS101 (0.1, 0.5, 1, 2 µg/ml) or LY294002 (50 µM). After 24 h, cell lysates were extracted for detection of PTEN, p-AKT, p-GSK3β, p-FoxO3a by western blot analysis, with actin as loading control. Results represent three experiments.

Increased proliferation is one of the first alterations mesangial cells undergo following exposure to high glucose concentrations [22]. To evaluate the efficacy of AS101 against this phenotype, we performed an XTT assay measuring viability of the cells followed by FACS analysis to determine cell cycle arrest. Mesangial cells treated with high glucose (30 mM) showed an increase in cell viability compared to serum depraved (starved; STR) and mannitol (MAN) controls (Fig. 4a). Treatment of HG combined with AS101 showed a significant dose dependent decrease in cell viability (significance achieved at 5 µg/ml) compared to cells treated with HG alone (Fig. 4a). In order to ensure that the effect was not due to toxicity of AS101, mesangial cells were treated with AS101 at different concentrations (0.1, 0.5, 1, 2, 5 µg/ml), which showed no significant effect on cell viability (Fig. 4b). Next, we evaluated cell cycle progression as a measure of mesangial cell proliferation. A larger percent of cells treated with HG entered the S phase compared to the osmotic (Mannitol) control (HG 34.11% vs. Man 26.1%). This HG-induced cell cycle progression was prevented by the addition of AS101 (HG 34.11% vs. HG+AS101 1 µg/ml 27.89%; 2 µg/ml 28.14%; 5 µg/ml 28.99%) (Fig. 4c). Moreover, mesangial cell expansion resulting from glucose-induced proliferation, was also decreased following AS101 administration (>0.5 µg/ml) (Fig. 4d). AKT and its downstream effectors have a pivotal role in cell viability and cell cycle progression. Mesangial cells treated with HG showed a higher level of phosphorylated AKT compared to control, corresponding to its activation (Fig. 4e). Adding AS101 at concentrations exceeding 0.5 µg/ml prevented HG induced AKT phosphorylation and maintained p-AKT level at comparable level to control cells (Fig. 4e). A common upstream down-regulator of AKT phosphorylation is PTEN, and its expression levels were decreased under HG conditions. AS101 addition (>0.5 µg/ml) prevented this downregulation, suggesting PTEN as an important mediator of the effects of AS101 (Fig. 4e). The downstream targets of AKT, GSK3β and FoxO3a also play a role in cell viability and cell cycle progression [15]. High glucose treated mesangial cells showed elevated levels of GSK3β and FoxO3a phosphorylation, corresponding to their inactivation (Fig. 4e). Addition of AS101 (>0.5 µg/ml) resulted in decreased phosphorylated levels of GSK3β and FoxO3a, similar to those of the controls (Fig. 4e). In addition, phosphorylation levels of AKT and GSK3β/FoxO3a were correlated (Fig. 4e) suggesting an AKT-mediated downstream effect on GSK3β and FoxO3a phosphorylation.

Pharmacologic inhibition of PI3K (LY294002) added to HG treated cells induced decreased levels of AKT, GSK3β and FoxO3a phosphorylation, while PTEN levels remained high (Fig. 4e). This pharmacologic inhibition was correlated with AS101 inhibition of AKT, GSK3β and FoxO3a phosphorylation, suggesting that AS101 and LY294002 might have a similar mechanism. In addition, AS101 and LY294002 had a similar phenotypic effect on mesangial expansion (Fig. 4d) which further substantiates a similar course of action.

### AS101 attenuates mesangial cell growth induced by high glucose

**Figure 5:**
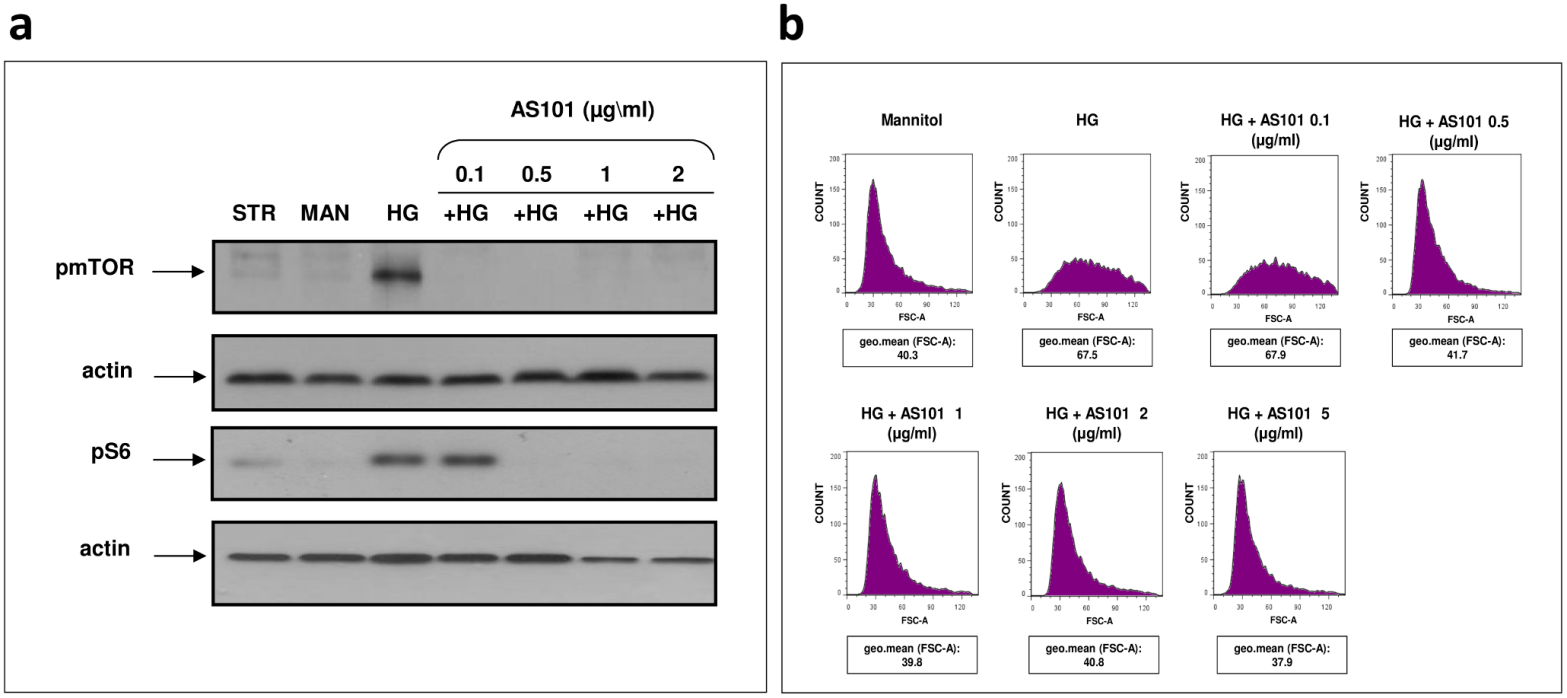
AS101 attenuates high glucose-induced mesangial cell growth. Mesangial cells were pre-treated as in figure 4. (a) Cells treated with HG were treated with different concentrations of AS101 (0.1–2 µg/ml). After 24 h, cell lysates were extracted for western blot analysis detection of p-mTOR; p-(rp)S6, with actin as loading control. Results represent three experiments. (b) Cells treated with HG were treated with different concentrations of AS101 (0.1–5 µg/ml). After 48 h of treatment, cells were stained with PI buffer. Cell size was determined by FACS, expressed in arbitrary units as assessed by Flowjo program under Forward Scattered analysis (FSC). Geometric mean (geo.mean) of cell size in each sample was determined. Results are representative of two experiments.

The potential role of AKT as a mediator in high glucose-induced mesangial cell proliferation, led us to investigate its downstream factors, which could possess a different kind of destructive effect. High glucose treatment resulted in the phosphorylation of mTOR on Ser2448, while osmotic (mannitol) and serum deprived (starved) controls did not exhibit any phosphorylation (Fig. 5a). Furthermore, AS101 decreased the level of p-mTOR in the presence of HG treatment (Fig. 5a). Moreover, the phosphorylation level of (rp)S6, a ribosomal protein located downstream to mTORC1 was also elevated under HG treatment, and was downregulated by the addition of AS101 to cell culture (Fig. 5a). This further establishes the inhibitory properties of AKT/mTOR axis by AS101 (Fig. 4e, 5a). Evidence suggesting mTOR as a key regulator in HG mediated mesangial hypertrophy [23] encouraged us to investigate the potential of AS101 to regulate mesangial cell hypertrophy under diabetic conditions. FACS analysis revealed high glucose-induced enlargement of mesangial cell size by ∼1.5 fold, as compared to osmotic control. This effect was eliminated when AS101 exceeding 0.5 µg/ml was supplied to HG treatment, maintaining cell size similar to control levels (Fig. 5b).

### AS101 abolishes collagen type 1 accumulation induced by high glucose in-vitro

**Figure 6:**
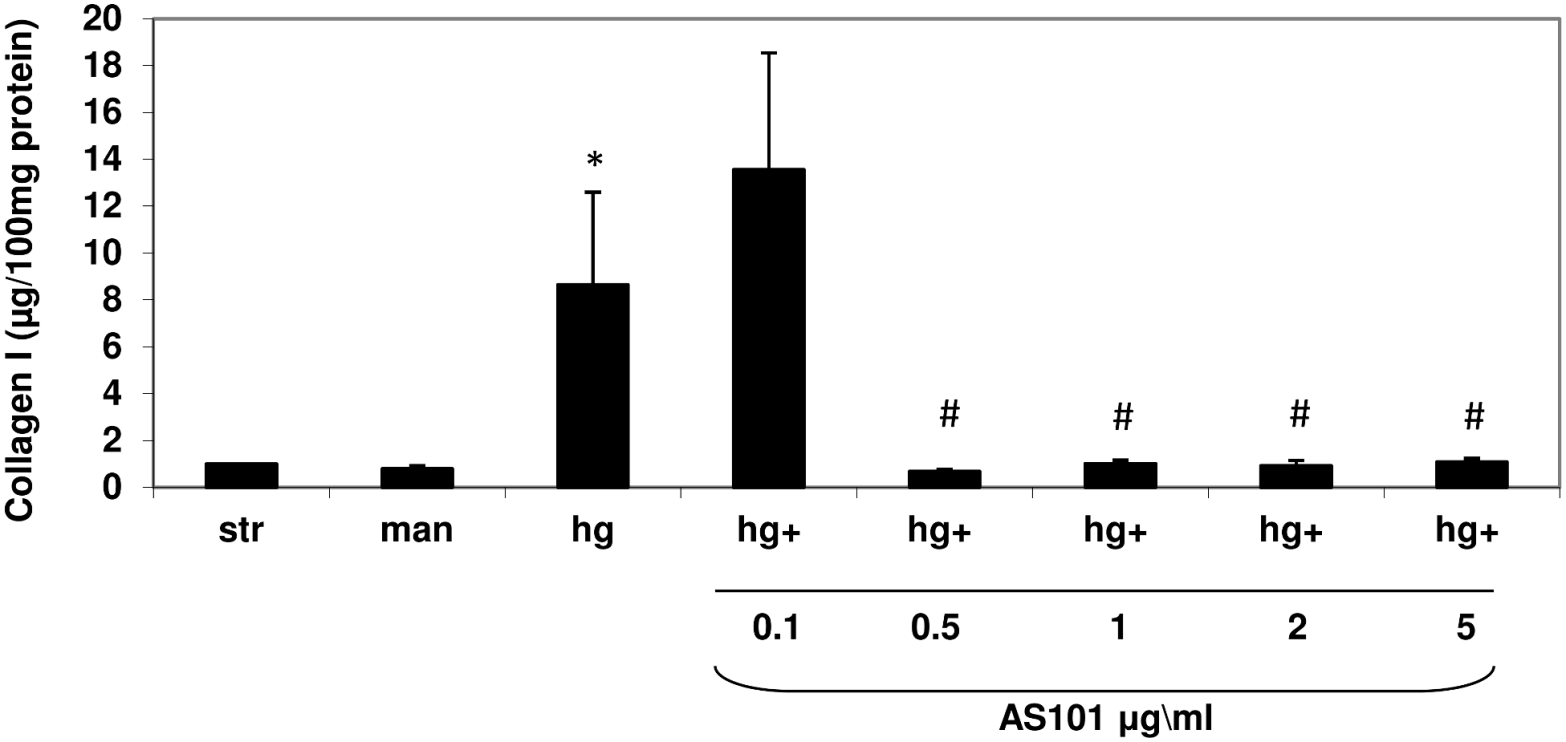
AS101 abolishes collagen type 1 production induced by high glucose in-vitro. Mesangial cells were pre-treated as in figure 4. Cells treated with HG were treated with different concentrations of AS101 (0.1–5 µg/ml). After 48 h, cell lysates were incubated with pepsin for collagen solubilization for 10–12 days. ELISA assay for the detection of collagen type I was performed on soluble collagen extracts. Results represent mean±SEM of four experiments. *p<0.05 increase vs. starved and mannitol groups. #p<0.05 decrease compared to HG group. Collagen in control cells (30.523 µg/100 mg protein) was normalized to 100%.

Previously, we demonstrated the ability of AS101 to maintain cell proliferation and cell size in a normal state under HG conditions. We next evaluated the effects of AS101 on ECM accumulation, which was previously suggested to be supported by the PI3K/AKT pathway [12]. Levels of collagen type 1 were evaluated by ELISA, which displayed increased collagen type 1 accumulation in HG treated mesangial cells compared to the controls (Fig. 6). In correlation with our previous results, addition of AS101 (>0.5 µg/ml) significantly downregulated HG-induced collagen type 1 accumulation (Fig. 6).

**Figure 7:**
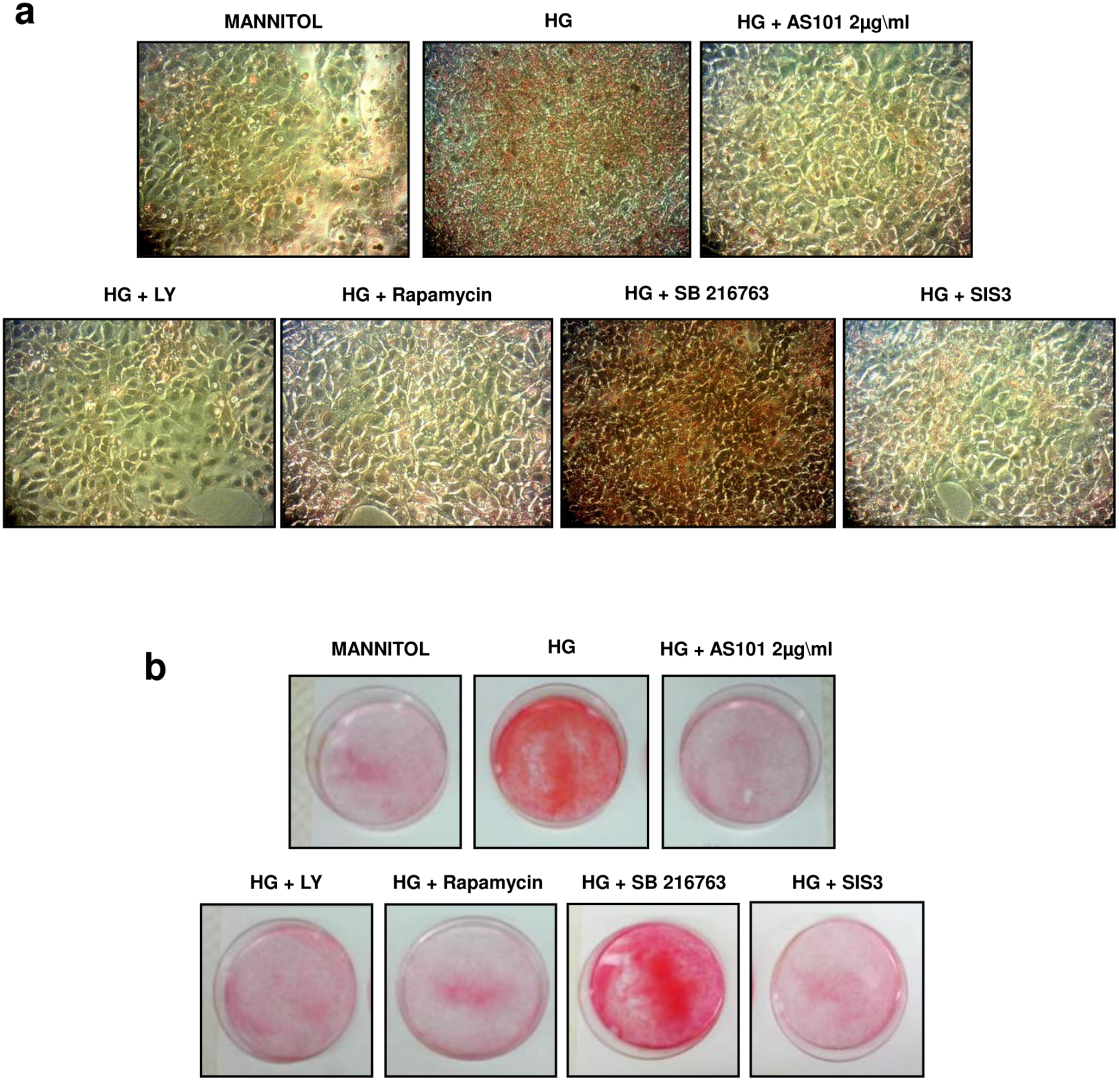
Effect of PI3K, mTOR, GSK3β and SMAD3 inhibitors on total collagen production in-vitro. Mesangial cells were pre-treated as in figure 4. (a, b) Cells treated with HG were separately treated with AS101 (2 µg/ml), LY294002 (50 µM), Rapamycin (0.1 ng/ml), PD98059 (100 µM), SB216763 (30 µM) and SIS3 (20 µM). After 48 h, cells were stained with Sirius red to detect collagen. Results are representative of two experiments. After washing the dye (a) Light microscope examination (X40) and (b) photograph of culture plates was performed.

Total collagen accumulation was estimated by Sirius Red staining. The staining was performed on mesangial cell cultures treated either with mannitol/starved controls or HG treatment. HG treated cells showed extensive total collagen staining compared to osmatic control (Fig. 7). AS101 treatment abolished HG-induced total collagen accumulation down to control levels (Fig. 7). These results suggest a beneficial effect of AS101 in downregulation of collagen accumulation, correlated with decreased AKT, mTOR, and GSK3β phosphorylation levels (Fig. 4e, 5a, 7).

### Effect of PI3K, mTOR, GSK3β, and SMAD3 inhibitors on total collagen production in-vitro

To determine the significance of PI3K, mTOR, GSK3β and SMAD3 activity in mediating HG-induced collagen accumulation, we added several pharmacological inhibitors to HG treated mesangial cells: the PI3K inhibitor (LY294002) and mTORC1 inhibitor (Rapamycin) each downregulated HG-induced total collagen accumulation, indicating a pivotal role for PI3K and mTOR in mediating this effect (Fig. 7). In addition, a SMAD3 inhibitor (SIS3) was added to HG cultured cells, which also resulted in decreased accumulation of total collagen (Fig. 7).

Consistent with these results, addition of the GSK3β inhibitor, SB216763, to HG cultured cells didn’t decrease HG induced total collagen levels (Fig. 7). This further establishes the role of GSK3β in SMAD3 degradation, since inhibition of GSK3β in HG treated cells resulted in increased collagen accumulation.

## Discussion

In this study, we demonstrate the effect of the tellurium based compound, AS101, in ameliorating Diabetic Nephropathy. Its effect was associated with downstream modifications of AKT mediated signal transduction, including enhanced mesangial cell viability, cell cycle progression, cell growth, and collagen type 1 accumulation. AS101 administration in-vivo resulted in downregulation of albuminuria, proteinuria and kidney hypertrophy. All of which were correlated with cortical downregulation of p-AKT, and p-AKT-mediated signal transduction.

One of the most highly recognized roles of AKT is its ability to promote cell cycle progression. Proliferation of mesangial cells is an early indicator of DN progression [20], and other studies have already suggested the role of PI3K/AKT in this process. Thus, for example, mesangial cells grown in HG and treated with LY294002, an inhibitor of PI3K, demonstrated less proliferation compared to HG treatment alone [24]. In addition, AKT phosphorylation levels after 24 h and 48 h correlated with HG-induced mesangial cell proliferation, which was decreased when a PI3K inhibitor was introduced to HG treated mesangial cells [25], [26]. Regarding AKT/FoxO3a;GSK3β signaling in apoptosis (hence viability), AKT has the ability to directly phosphorylate and deactivate BAD, a pro-apoptotic factor [27], or phosphorylate and deactivate its downstream effectors, GSK3β and FoxO3a, which contribute to the pro-apoptotic state by deactivating anti-apoptotic factors, such as MCL-1, or activating pro-apoptotic factors, like BIM [28], [29].

In addition to the role of PI3K/AKT in promoting cell viability, it also plays a role in cell cycle progression and proliferation. AKT phosphorylates and deactivates p27^Kip1^ and p21^cip1/WAF1^, which are known to inhibit cell cycle progression, thereby enhancing cell proliferation [30], [31]. In addition, protein levels of p27^Kip1^ are downregulated via the inhibition of its transcription factor FoxO3a which can also promote cell cycle progression [32]. On the other hand, GSK3β phosphorylation by AKT prevents it from phosphorylating and degrading Cyclin D/E and c-myc/c-jun which contribute to cell cycle progression, leading to cell proliferation [33]–[36].

Under diabetic conditions, AKT/FoxO3a phosphorylation levels were increased in kidney cortex of diabetic rats after 2 weeks of STZ injection suggesting an early involvement of AKT/FoxO3a in the onset of DN. In addition, AKT induced FoxO3a inactivation under TGFβ stimulation caused mesangial cell survival [29]. Therefore, there is a significant role of downstream effectors FoxO3a [37] and GSK3β [38] in hyperglycemic modulation of mesangial cell dysfunction.

A different regulator in hyperglycemic mesangial dysfunction is mTOR and its downstream effectors play a crucial role in cell growth and hypertrophy [39]. The importance of the mTORC1 complex was demonstrated in diabetic renal pathology, and rapamycin is experimentally used as a therapeutic agent against the progression of diabetic nephropathy [23], [40], [41]. The AKT/mTOR pathway plays a role in HG-induced mesangial cell proliferation [42], as well as mesangial and glomerular hypertrophy [43]. Moreover, inhibition of mTOR by rapamycin downregulates these effects [42], [43]. In fact, rapamycin is sufficient to arrest HG induced mesangial proliferation [42]. This could be explained by the oncogenic role of mTOR, mediating the translation of mRNAs that encode cyclin D and c-myc proteins [44], demonstrating an additional pathway by which mTOR functions as a cell cycle activator. In this case, both the inhibition of AKT or mTOR can lead to cell cycle arrest that prevents the next stage of hypertrophy.

Here, we show phosphorylation of AKT and mTOR following incubation for 24 h under HG conditions; this effect was followed by cell cycle progression and cell size enlargement after 48 h. These conditions resulted in S phase difference between HG treated cells and mannitol treated cells of 8% combined with a ∼1.5 fold difference in the average cell size. The fact that cell cycle progression occurs while cell size increases might be explained by experiment conditions that “caught” the cells in a transition state between cell cycle arrest and hypertrophy. This combined HG-induced cell enlargement and cell cycle progression was successfully inhibited by AS101, which also prevented AKT/mTOR downstream signaling. Our in-vivo studies showed that decreased hypertrophy of whole kidney was significant only at High Dose (1 mg/kg) of AS101 administration. In addition, AS101 at both doses (0.5 mg/kg; 1 mg/kg) successfully downregulated the phosphorylation of AKT. Increased levels of p-AKT has already been identified in diabetic animals [40], [43], [45], [46], linking PI3K/AKT/mTOR signal transduction in cortical tissue to the progression of renal hypertrophy [40], [43], [46]. Proliferation of mesangial cells in glomeruli of diabetic mice was also correlated with AKT phosphorylation [46], suggesting that AKT has an important role in kidney dysfunction under diabetic conditions.

In DN, the most important glomerulus alteration leading to proteinuria and kidney failure is basal membrane matrix deposition. Early indications suggested a pivotal role of PI3K/AKT in regulation of fibroblast laminin and collagen type 4 expression [47]. In addition PI3K/AKT regulates human lung fibroblast alpha 1(I) mRNA stabilization, hepatic stellate cell proliferation, and collagen type 1 expression [48], [49].

Thus, PI3K/AKT signaling emerged as a significant mediator of matrix modulation and has a specific role in HG induced primary rat mesangial cell collagen type 1 transcription and translation [13]. In addition, the well-known TGFβ/SMAD3 fibrotic signal was also found to be mediated by PI3K/AKT signaling, which further establishes the importance of this pathway in HG mediated glomerulosclerosis [12].

The correlation between AKT phosphorylation state and collagen production led us to investigate its downstream effect in this process. AKT phosphorylates its downstream effector GSK3β, leading to its inactivation. This downstream signaling of AKT/GSK3β was also suggested to contribute to TGFβ stimulated SMAD3 stability in a variety of cell lines [50], emphasizing the importance of GSK3β in collagen accumulation. Downregulation of AKT phosphorylation by AS101 contributes to decreased collagen expression by mesangial cells. We assume that AS101 prevents AKT from phosphorylating SMAD3, thus keeping it outside of the nuclei [12] which results in prevention of collagen accumulation. In addition, we demonstrate that when PI3K, mTORC1, and SMAD3 are each pharmacologically inhibited in HG treated mesangial cells, collagen accumulation decreases, while inhibition of GSK3β does not affect total collagen levels. This is correlated with the ability of AS101 to reduce collagen levels, further substantiating our hypothesis that AS101 downregulation of collagen might be attributed to the AKT downstream signaling modulation.

Furthermore, our study showed that AS101 administration in-vivo decreased kidney dysfunction combined with the downregulation of AKT, GSK3β and SMAD3 phosphorylation in kidney cortex of diabetic rats. Previous studies showed presence of activated AKT in glomeruli of diabetic rats [13], which correlated with collagen type 1 accumulation in-vivo [14]. Thus, it is possible that modulation of AKT/GSK3β/SMAD3 by AS101 which leads to attenuation in collagen expression plays a protective role against the development of renal dysfunction.

In this study, we show the pharmacological properties of AS101 in mitigating the progression of DN. We believe that the compound’s ability to prevent HG-induced glomerulosclerosis by preventing insult to mesangial cells leads to kidney protection. Here, we focused our study in the ability of AS101 to downregulate AKT as a central mechanism of action leading to normalize cell viability, proliferation, cell size and collagen deposition.

Available therapies, including adequate glycemic control and anti-hypertensive therapy, only slow down but do not halt the progression of renal dysfunction. AS101 is a non-toxic compound, currently in phase 2 clinical study and has a variety of additional clinical applications. Here, we suggest AS101 as a potential therapeutic agent against the progression of DN. Moreover, growing evidence of the significance of PI3K/AKT pathway in lung [51] and liver fibrosis [52] might lead us to a broader clinical significance for tellurium based compounds in the future.

## Materials and Methods

### Materials

Reagents, inhibitors and antibodies and their sources were as follows: cell culture media, penicillin/streptomycin, L-glutamine, fetal bovine serum (Biological industries, Beit Haemek, Israel); D-(+)-Glucose, Propidium iodide, Picro-sirius Red and Actin HRP antibody (Sigma, Rehovot, Israel). Streptozotocin, D-(+)-mannitol and pharmacologic inhibitores LY294002, PD98059, SB216763, SIS3 (Calbiochem; Dermstadtt, Germany). Rapamycin inhibitor (LC laboratories, Woburn, MA, USA). Antibodies: p-AKT (Ser473), PTEN, p-(rp)S6 (Ser235/236), p-mTOR (Ser2448), p-FoxO3a (Thr32), p-GSK3β (Ser9) (Cell Signaling, Denvers, MA, USA). p-SMAD3 (Ser425) antibody (Santa Cruz Biotechnology, CA, USA). Rat Type I Collagen detection kit (Chondrex, NE Redmond, WA, USA). Cell proliferation kit (XTT) (Biological industries, Beit Haemek, Israel).

### Animal studies

This study was carried out in strict accordance with the recommendations in the Guide for the Care and Use of Laboratory Animals of the National Institutes of Health. The protocol was approved by the Institutional Animal Care and Use Committee of Bar-Ilan University (Permit Number: 8-03-08), and all efforts were made to minimize suffering. Diabetes was induced in 53 male Sprague-Dawley rats weighing approximately 200-gr (Harlane laboratories, Jerusalem, Israel) with single 60 mg/kg streptozotocin (STZ; Calbiochem) injection into the tail-vein. The control group received vehicle injection (0.1 M citrate buffer, pH 4.5). The induction of diabetes was confirmed by blood glucose monitoring 3 days after the STZ injection and only rats with blood glucose above 300 mg/dl were considered diabetic. Rats were monitored every two weeks for weight and blood glucose. AS101 was introduced by single intra-peritoneal injection at dose of 0.5 mg/kg (low dose) or 1 mg/kg (high dose) in 1 ml PBS every other day. The control group was treated by 1 ml PBS at the same regime. The rats were placed in metabolic cages and urine was collected one day before diabetes induction, at two weeks and at four weeks. At four weeks the rats were sacrificed by carbon dioxide asphyxiation, kidneys were collected, weighted and trunk blood was collected. Kidney cortex was separated, snap-frozen in liquid nitrogen and homogenized in PBS. The homogenized tissue was washed twice by centrifugation (3000 g at 4° degree) and protein analysis was done as described above.

### Cell culture

Rat mesangial cells were obtained from a culture of glomeruli isolated from male Sprague-Dawley (SD) rats using differential sieving methods as described previously [53]. Isolated glomeruli were cultured in Dulbecco’s modified Eagle’s medium (DMEM) containing 5.5 mM D-(+)-glucose; 20% Fetal Bovine Serum (FBS), 100 µg/ml streptomycin, 100 µg/ml penicillin, and 2 mM L-glutamine, at 37°C in an atmosphere containing 5% CO_2_. Cells were trypsinized for passage with 0.05% Trypsin in 1 mM EDTA. Cells were transferred every 48 h when 80% confluence was achieved. At the beginning of each experiment, cells were cultured for 24 h in DMEM containing 100 µg/ml streptomycin, 100 µg/ml penicillin, 5.5 mM D-(+)-Glucose, and 2 mM L-glutamine, followed by 24 h or 48 h treatment as described.

### HG treatment

After 24 h of incubation, cells were transferred to fresh mesangial cell culture medium containing 5.5 mM D-(+)-Glucose, with an addition of 24.5 mM D-(+)-Glucose for the total of 30 mM D-(+)-Glucose (High glucose concentration) (Sigma) Control cultures were supplied with 24.5 mM Mannitol (osmotic control) (Calbiochem) or maintained in DMEM containing 5.5 mM D-(+)-Glucose. In addition, HG treated cells were treated with different concentrations of AS101 (0.1, 0.5, 1, 2, 5 µg/ml) or pharmacologic inhibitors: 50 µM LY294002, 0.1 ng/ml Rapamycin, 100 µM PD98059, 30 µM SB216763, and 20 µM SIS3 (Calbiochem), as indicated for each experiment. All treatments lasted for 24 or 48 hours, as indicated.

### Cell count

Mesangial cells were trypsinized from culture plates, centrifuged and suspended in DMEM. Then, a sample from cell suspension was diluted 1∶1 with Trypan Blue dye (0.4%) and counted in a hemocytometer under a light microscope.

### XTT assay

Cells (5×10^4^/well) were cultured in 96 well plates. After 24 h incubation in DMEM without serum, treatments were added to each plate, as indicated. After 24 h of treatment, 50 µl of XTT reagent was added to each plate according to the manufacturer’s protocol (Biological Industries) for 2–5 h following solubilization, the orange color was detected by spectrophotometer at 450 nm.

### Western blot analysis

After 24 h of treatment, mesangial cells were lysed with lysis buffer (1 M Tris (pH = 7.4), 1.5 M NaCl, 1% Triton-X, 10% Glycerol, 50 mM EDTA (pH = 8), 0.1 M Sodium vanadate, 0.1 M PMSF, 0.1% protease inhibitor cocktail (Calbiochem) Samples were boiled for 5 minutes, electrophoresed on 8% or 12% SDS-PAGE, transferred to nitrocellulose, and immunoblotted with specific Antibodies (p-AKT, p-GSK3β, p-FoxO3a, PTEN, p-(rp)S6 and p-mTOR, (Cell Signaling) actin HRP (Sigma). Blots were developed using horseradish peroxidase-conjugated secondary Abs and the ECL detection system (Thermo scientific, Pierce research protein products (Rockford, IL, USA)).

### Cell cycle progression and cell size analysis

Propidium Iodide (PI) solution (Sigma) was diluted in double-distilled water (ddH_2_O) at 4°C, protected from light. PI buffer contained 0.1% Triton X-100, 0.1% Sodium Citrate, 50 µg/ml PI in Dulbecco’s Phosphate buffered saline (PBS) without calcium or magnesium and was also protected from light. After treatment, mesangial cells were trypsinized and washed twice with PBS (w/o Ca^++^; Mg^++^). The cell pellet was suspended in 500 µl of PI buffer and 1 mg/ml of RNAse (Sigma). After 40 minutes at 4–8°C in the dark, cell aggregates were removed by silk fabric filtration. Then, DNA content was evaluated for cell cycle progression using FACS flow cytometer and the Flowjo cell cycle analysis program. Cell size was evaluated by FACS analysis of FSC-A (forward scattered) parameter of each sample.

### Sirius Red staining

After 48 h treatment, mesangial cell culture medium was extracted, and cells were washed twice with PBS while attached to the plate surface. Next, Picro-sirius Red stain containing 0.1% Sirius red F3B diluted in Picric acid was added to the attached cells for 1 h. Then, cell cultures were washed twice with acetic water (0.5% Acetic acid (glacial) in ddH_2_O) followed by two washes with tap water. The red stained collagen protein was detected under a light microscope (X40) or by visual inspection.

### Collagen type 1 detection

After 48 h of treatment, mesangial cells were scraped from culture plates and transferred to collagen solubilization tubes coated with NGS buffer (Normal goat serum (NGS) (Biological Industries)/0.1 Tris-base/0.15 M NaCl/pH = 7.5). Mesangial cells were pre-treated for collagen type 1 detection by solubilization: Cells were suspended in 0.1 mg/ml pepsin diluted in 0.05 M acetic acid at 4°C for 24–48 h repeated 5 times. At the end of each solubilization, cells were centrifuged and supernatant was preserved. After five cycles, cells were centrifuged and suspended in 0.1 mg/ml pancreatic elastase in 0.1 M Tris, 0.15 M NaCl, 5 mM CaCl_2_ for 24 h at 4°C, after which cells were centrifuged and supernatant was saved. Supernatant containing soluble collagen from all the solubilization cycles was further analyzed for Rat collagen type 1 by ELISA kit (Chondrex Inc., Redmond WA, USA). To determine protein content in each sample a Bradford protein assay was performed (Bio-Rad, CA, USA), and collagen type 1 was evaluated per 100 mg protein per sample.

### Statistics

Results shown in this study, expressed as means ±S.E. *p*<0.05 was considered statistically significant. Differences in clinical symptoms between groups were analyzed using one-way ANOVA test. Differences between groups in XTT viability assay and Collagen type 1 detection assay were analyzed using Student’s *t* test.
